# A perfusion incubator liver chip for 3D cell culture with application on chronic hepatotoxicity testing

**DOI:** 10.1038/s41598-017-13848-5

**Published:** 2017-11-06

**Authors:** Fang Yu, Rensheng Deng, Wen Hao Tong, Li Huan, Ng Chan Way, Anik IslamBadhan, Ciprian Iliescu, Hanry Yu

**Affiliations:** 1 Institute of Bioengineering and Nanotechnology, A*STAR, The Nanos, 04-01, 31 Biopolis Way, Singapore, 138669 Singapore; 2 NUS Graduate School for Integrative Sciences and Engineering, Centre for Life Sciences (CeLS), 28 Medical Drive, Singapore, 117456 Singapore; 3 MechanoBiology Institute, National University of Singapore, T-Lab, 5 A Engineering Drive 1, Singapore, 117411 Singapore; 4 Department of Physiology, National University of Singapore, MD9 03-03, 2 Medical Drive, Singapore, 117597 Singapore; 5 Singapore-MIT Alliance for Research and Technology, 1 CREATE Way, #10-01 CREATE Tower, Singapore, 138602 Singapore; 6 MechanoBiology Institute, National University of Singapore, T-Lab, 5 A Engineering Drive 1, Singapore, 117411 Singapore; 7 National Institute for Research and Development in Microtechnologies, IMT-Bucharest, Bucharest, 077190 Romania; 8 Academy of Romanian Scientists, Splaiul Independentei nr. 54, sector 5, Bucharest, 050094 Romania; 9 Bigheart, National University of Singapore, MD6, 14 Medical Drive, #14-01, Singapore, 117599 Singapore

## Abstract

Liver chips have been developed to recapitulate *in vivo* physiological conditions to enhance hepatocyte functions for assessing acute responses to drugs. To develop liver chips that can assess repeated dosing chronic hepatotoxicity, we need to ensure that hepatocyte functions be maintained at constant values over two weeks in stable culture conditions of sterility, temperature, pH, fluidic-flow of culture media and drugs. We have designed a perfusion-incubator-liver-chip (PIC) for 3D cell culture, that assures a tangential flow of the media over the spheroids culture. Rat hepatocyte spheroids constrained between a cover glass and a porous-ultrathin Parylene C membrane experienced optimal mass transfer and limited shear stress from the flowing culture media; maintained cell viability over 24 days. Hepatocyte functions were significantly improved and maintained at constant values (urea, albumin synthesis, and CYP450 enzyme activities) for 14 days. The chip act as an incubator, having 5% CO_2_ pressure-driven culture-media flow, on-chip heater and active debubbler. It operates in a biosafety cabinet, thus minimizing risk of contamination. The chronic drug response to repeated dosing of Diclofenac and Acetaminophen evaluated in PIC were more sensitive than the static culture control.

## Introduction

‘Liver-on-a-chip’ models have attracted much attention since they recapitulate some *in vivo* tissue structures and functions, biochemical cues and mechanical microenvironment. These models permit the study of drug interactions *in vitro* and could be alternatives to animal experimentation^1,2^. Liver chip models utilize minimal cells and reagents, allowing more tests to be performed using human cells of limited quantity. The most common ‘Liver-on-a-chip’ models for drug testing are for testing acute drug toxicity^3–5^. They have not been adapted to long-term cell culture and chronic toxicity testing, due to the more pronounced problems of contamination, clogging and bubble accumulation in the chips that deteriorate cell functions over extended culture period of weeks^6,7^.

Contamination of bacteria, mycoplasma or fungi is a common problem in long-term cell culture. Two stages are critical in ensuring sterility in long-term cell culture: 1) initial chip assembly and cell seeding, 2) exposure to contaminants during the extended cell culture over weeks. There are a few strategies to prevent contamination in the stage 1 of chip assembly, such as high temperature treatment or autoclave, Gamma irradiation or UV treatment, and filtration of fluid^8,9^. Proper sterile techniques in primary cell isolation from animals can ensure sterility of the seeded cells in the chips^10^. Stage 2 is more problematic if we need to repeatedly disconnect and reconnect the media/drug reservoir to the cell-containing chips. For monitoring cell responses under a microscope, we also frequently move the chips in and out of the incubator. To minimize such frequent movements and connect/disconnect in the non-sterile incubators, we integrate all the perfusion chip accessories into a single perfusion incubator chip (PIC) such that the entire cell culture process is performed in the sterile biosafety cabinet.

In long-term microfluidic culture systems, dissociated gas from the culture media often results in bubble accumulation in the microfluidic channels. This leads to clogging and dead volumes. Bubble traps commonly used in microfluidic systems can prevent large bubbles from entering the culture chamber; however, they cannot prevent the formation of dissociated gas or tiny bubble accumulation inside the culture chambers. Thus, we have integrated an active debubbler in the PIC to remove bubbles from the chamber in real time.

A proper design of a chronic liver toxicity-testing chip should have the following features:

- Organotypic 3D cellular architecture^11–15^;
- Good mass transfer, allowing diffusion of O_2_ and nutrients from media to the spheroids and removing the metabolites and by-products from the proximity of the spheroids^1,16–18^;
- Maintenance of mechanical forces and limited shear stress to the cells^1,7,18^;
- Stable cell culture conditions: temperature, pH, sterility and optimized culture media composition^1,7,18^;
- Ease of handling cells and replenishing media^7^;

Here we report a method and device for long-term 3D hepatocyte culture, which maintains the cell viability over 3 weeks and functions over 2 weeks, and its application on chronic hepatotoxicity testing. An essential aspect is that the design of the device assures a tangential flow over the cell culture, removing the metabolites and by-products from the proximity of the cells and refreshing the cell environment. A constrain spheroids 3D rat hepatocytes cell culture model was used for the testing. In this model, ultrathin Parylene C porous membrane constrained the hepatocyte spheroids formed underneath; it protects the hepatocytes from the shear stress while its relatively large pore size (20 µm) assures a good mass transfer. The membrane also immobilises the spheroids and prevents them from colliding into each other, which frequently happens on non-adhesive surfaces^19^ and ligand-modified films^20^. PIC with integrated temperature, pH and bubble control provides contamination-free and clogging-free environment to maintain rat hepatocytes viability and metabolic activities for 2–3 weeks. We applied the PIC for acute and chronic repeat dosing drug safety testing using two model-drugs diclofenac and acetaminophen (APAP). PIC addressed the various issues affecting the robustness and performance of liver chips for chronic drug testing.

## Results

### PIC Structure and Functionality

#### Device structure and microfluidic setup

**Figure 1:**
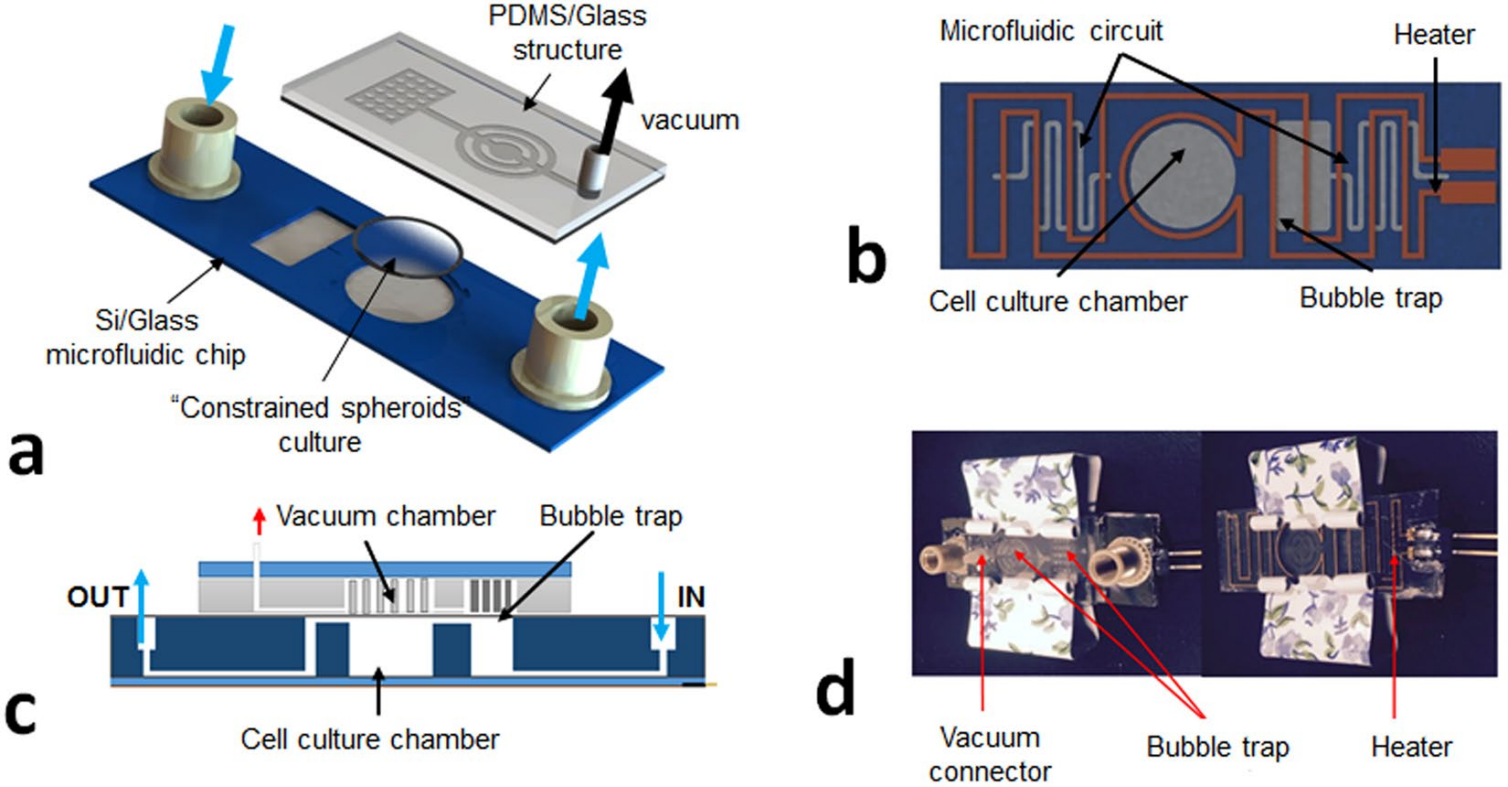
Schematic of the PIC chip. (**a**) 3D view with the PIC. A glass/silicon structure containing a 3D microfluidic circuit, the cell culture chamber, a bubble trap chamber as well as a heater (**b**) bottom view of the chip’s layout illustrating the microfluidic circuit, the cell culture chamber, the bubble trap and the heater, (**c**) cross-section of the PIC illustrating the structure of the bubble trap. It consists of a 70 µm-thick PDMS membrane (gas permeable) bonded to a PDMS molded chamber with pillars that support the membrane. The PDMS structure is connected to external vacuum (through a pressure controller). The gas bubbles trapped in the microwell can diffuse through the PDMS membrane due to negative pressure in the vacuum chamber while culture media remains inside the culture chamber. (**d**) Top and bottom view of the PIC.

The perfusion incubator liver chip (PIC) was designed combining a rigid, well defined and reusable glass/silicon structure with the advantages of PDMS assemblies (elasticity and gas permeable). The chip structure consists of three main elements illustrated in Fig. 1a:

- A glass/silicon structure containing a 3D microfluidic circuit, the cell culture chamber, a bubble trap chamber and a heater (Fig. 1b). Nanoport microfluidic connectors were mounted on the glass/silicon die. The microfluidic circuits were engraved on both sides of silicon die while the glass die assures the sealing of the bottom microfluidics circuit. On the other side of the glass die a heater is printed.
- A cell culture support (in our case a 100 µm-thick glass coverslip and a Parylene C membrane mounted on a Silicon ring). The constrained spheroid structure can be inserted and removed from the culture chamber.
- A PDMS/glass sealing structure closes the microfluidic circuit. The PDMS structure also acts as a debubbler to absorb the air bubbles trapped when assembling the chip and generated during the culture. A cross-section of the chip illustrating the functions of the PDMS/glass die is presented in Fig. 1c.

The main materials of the PIC are silicon and glass. The main advantage is their biocompatibility^21,22^, while having a hydrophilic surface of the microfluidic elements, there is less absorption of proteins and drug molecules. This is an important aspect for drug screening applications, especially when working with lower drug concentration^23^. The silicon structure assures a uniform temperature of the media, due to its good thermal conductivity. Moreover, the device can be reused to generate reproducible experimental results; and can be sterilized by flowing ethanol. For testing purposes, the silicon/glass structure was fabricated with different depths of the cell culture chamber (0.5 mm, 1 mm, 2 mm and 3 mm). When the cell culture chamber is clamped with a soft deformable PDMS cover, the high rigidity of silicon enables water-tight sealing of the microwell.

To minimize the risk of microbial contamination, the PIC was kept inside a biosafety cabinet at all times. To achieve this, we integrated a heater on chip. The heater was connected in series with a temperature controller and a thermocouple. Using a second thermocouple, the temperature of the cell culture media in the cell culture chamber and at the outlet was calibrated. The measured temperature is continuously adjusted to the set point of the temperature controller. The integration of the heater on the chip avoids the use of the incubator. Pressurized CO_2_ was used to drive media perfusion through the chip (Supplementary 1). CO_2_ dissolves in water forming carbonic acid, which keeps pH of the media constant.

We integrated bubble trap and active debubbler on chip to remove bubbles accumulated in the culture chamber. We avoided the use of external bubble traps to reduce the complexity of the system (more fluidic connectors) and surface area in contact with the cell culture media. Cells exposed to air bubbles are subjected to higher shear stress due to the stretching force at liquid-air interface. Large bubbles can cause cell death. As shown in (Fig. 1c), the debubbler consists of a 70 µm-thick PDMS membrane (gas permeable) bonded to a PDMS molded chamber with pillars that support the membrane. The PDMS structure is connected to external vacuum (through a pressure controller). Gas bubbles trapped in the microwell can diffuse through the PDMS membrane due to negative pressure in the vacuum chamber while culture media remains inside the culture chamber. Time-lapse images show that the vacuum eliminated the bubbles inside the microwell in 30 min (Supplementary 2).

#### Fabrication of microfluidic glass/silicon structure

**Figure 2:**
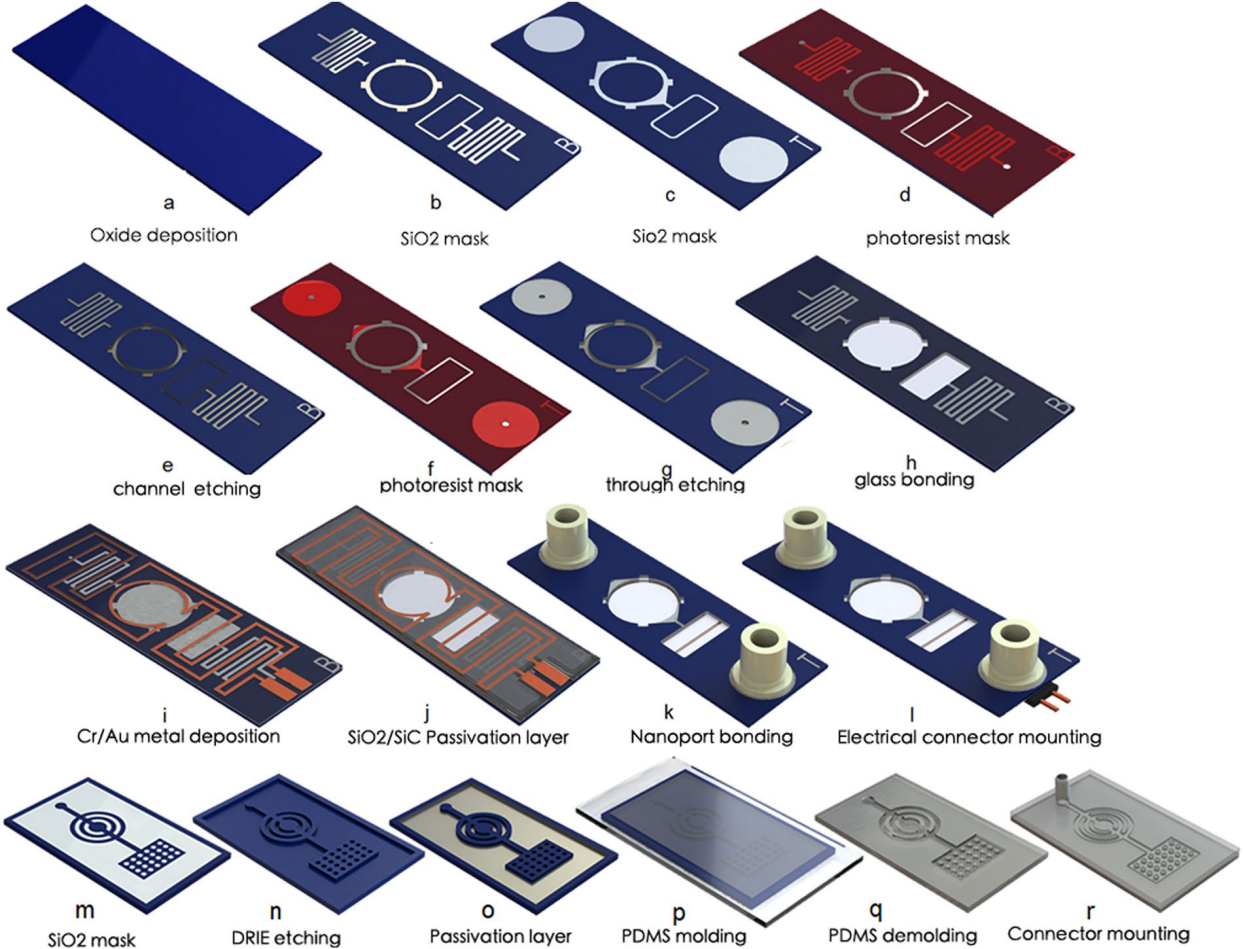
Main steps of the PIC fabrication process: (**a**–**l**) fabrication of the Si/glass structure (legend T = top of the wafer, B = Bottom of the wafer) (**m**–**r**) fabrication of the PDMS/glass cover.

The glass/silicon structure was fabricated in four versions with varying depth of the cell culture chamber (0.5 mm, 1 mm, 2 mm and 3 mm). We will describe the fabrication process for the device with the depth of the chamber of 1mm. A 4” silicon wafer, 1mm-thick, with the crystallographic orientation <100> was used for the fabrication of the microfluidic circuits. Main steps of the fabrication process are illustrated in Fig. 2. A similar process is described in^23^.

The silicon wafer was cleaned in piranha (H_2_SO_4_/H_2_O_2_ in ratio 2/1) in a quartz tank at 120 °C for 20 min, rinsed in DI water and spun dried. A 2 µm-thick thermal SiO_2_ layer (wet process) was grown on the Si surface in a furnace (Tystar, USA) at 1050 °C for 11 hours (Fig. 2a). The pattern with the microfluidic circuit was transferred on the SiO_2_ layer (bottom side of the wafer) using a 2 µm-thick photoresist mask (AZ7217-Clariant) and a classical RIE etching process in CHF_3_/O_2_ using and (SPTS reactor) – Fig. 2b. After the RIE process, the photoresist mask was removed in an ultrasonic tank with NMP at 70 °C for 30 min, rinsed in DI water and spun dry. The opposite microfluidic circuit was aligned and imprinted in a similar manner on the other side of the Si wafer (Fig. 2c). A 10 µm-thick photoresist mask, having the pattern of the etch-through features (inlet outlet holes, via-holes, cell culture chamber and bubble-trap chamber) was aligned and applied on the bottom of the wafer. Through this mask, trenches were etched in Silicon (400 µm in depth) using a classical Bosch process (on an Alcatel 101SE ICP Deep RIE) (Fig. 2d). The photoresist mask was striped using O_2_ plasma process in the same equipment, the SiO_2_ mask was then revealed. Another anisotropic etching of silicon was performed through the SiO_2_ mask (100 µm deep). It generated the microfluidic circuit on the bottom of the Si wafer (Fig. 2e). A Teflon layer was uniformly deposited in the Deep RIE system using C_4_F_8_ chemistry for protection of the microfluidic structure during deep RIE etching from the opposite side of the wafer. In the next step (Fig. 2f), the process described in Fig. 2d (400 µm deep trenches using Bosch process through a photoresist mask) was repeated, this time from the topside of the wafer. After removing the photoresist mask in O_2_ plasma and temporary bonding onto a dummy Si wafer with wax^24^, the microfluidic circuit from the top side of the wafer was defined using the same Bosch process till etch-through holes were completed (Fig. 2g). After wax removing in NMP ultrasonic tank and rinsing with DI water, the remaining SiO_2_ masks (top and bottom side of the wafer) were removed in BOE (Buffer Oxide Etch) solution, rinsed in DI water and cleaned in piranha. A 200nm-thick dry SiO_2_ layer was grown on the surface of Si wafer in a furnace. This SiO_2_ layer assures a hydrophobic surface of the microfluidic structure. The silicon wafer is then anodically bonded on a 4”, 500 µm-thick Pyrex glass wafer (Corning 7740) (Fig. 2h). The heater of the chip consists of a Cr/Au metal deposition (40 nm/1 µm)(CHA e-beam evaporator). It was defined using a classical photolithographic process (AZ7217) and Cr/Au wet etching solutions (Fig. 2i). A passivation layer SiO_2_/SiC (PECVD) was deposited on the heater and patterned using a photolithographic process and RIE process (Fig. 2j). This layer assures electrical isolation and chemical protection of the heater. Finally, the wafer was diced. Nanoport microfluidic connectors (Upchurch) (Fig. 2k) and an electrical connector were mounted on the chip (Fig. 2l).

### PIC System optimization

#### Flow simulation

**Figure 3:**
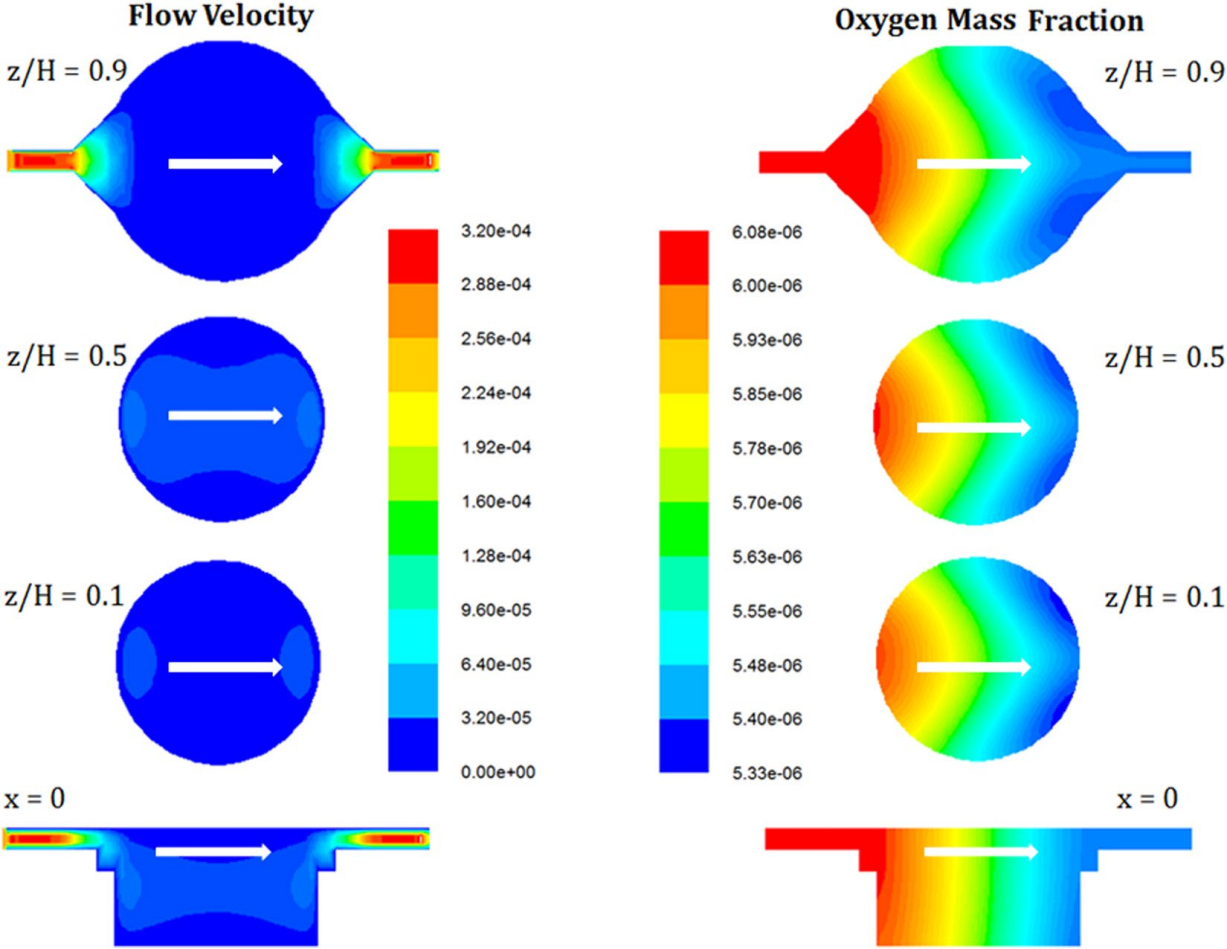
Simulation of the flow velocity and O_2_ concentration at different depths of the cell culture chamber. The shear stress is uniform in the cell culture chamber due to the large cross-sectional area of the bioreactor. This may help to lower the shear stress on the cells under the porous membrane. The O_2_ concentration decreases along the flow direction due to the O_2_ consumption by the cells.

Figure 3 shows the profiles of flow velocity and O_2_ concentration at different depths in the cell culture chamber. Although the velocity is high near the inlet and outlet, it becomes relatively low at the membrane plane due to the large cross-sectional area of the cell culture chamber. This may help to lower the shear stress on the cells under the porous membrane. The O_2_ concentration decreases along the flow direction due to the O_2_ consumption by the cells at the bottom.

**Figure 4:**
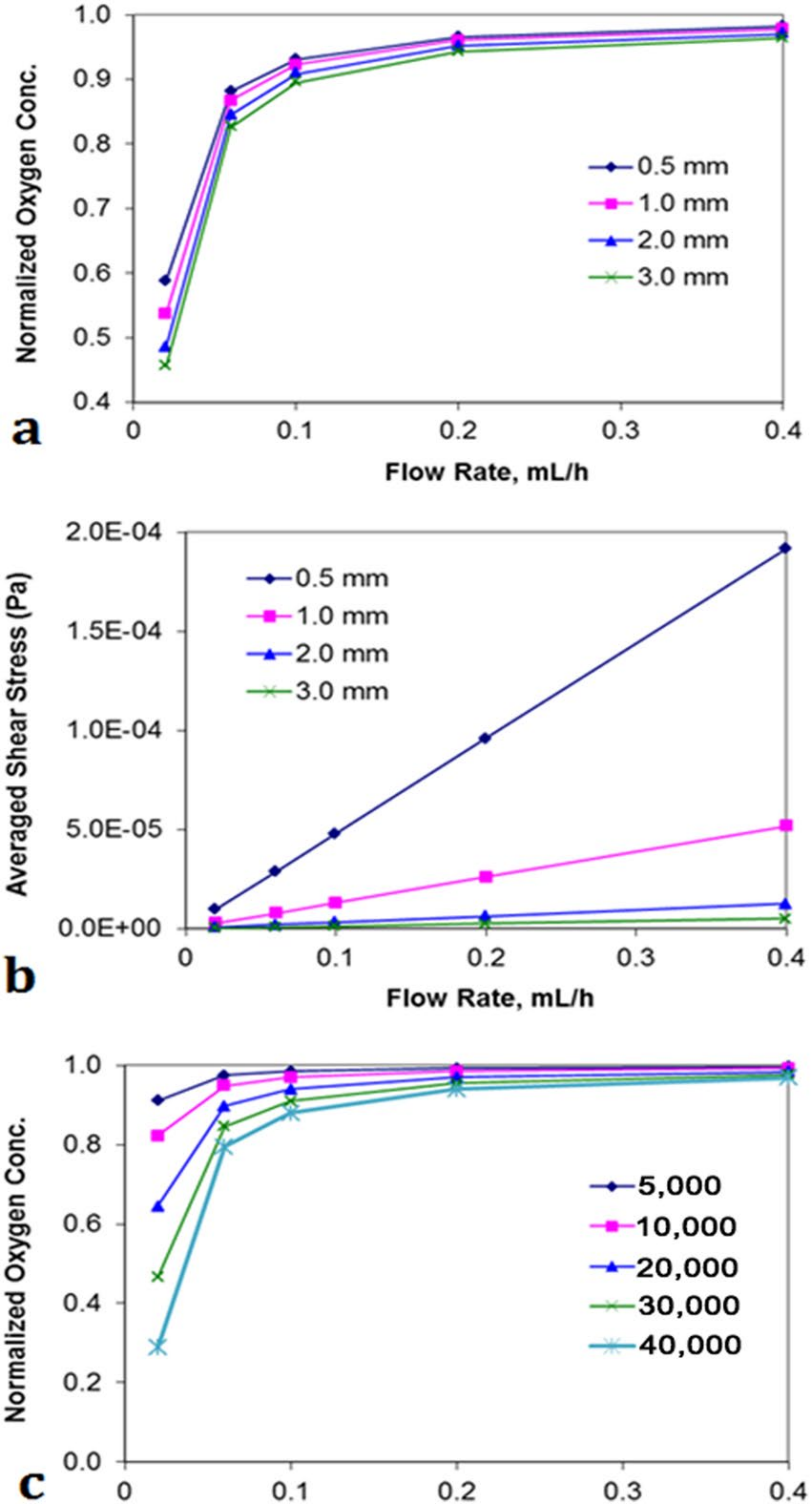
CFD simulations (**a**) oxygen concentration (**b**) average of shear stress (**c**) normalized oxygen concentration for different depth of the cell culture chamber.

**Figure 5:**
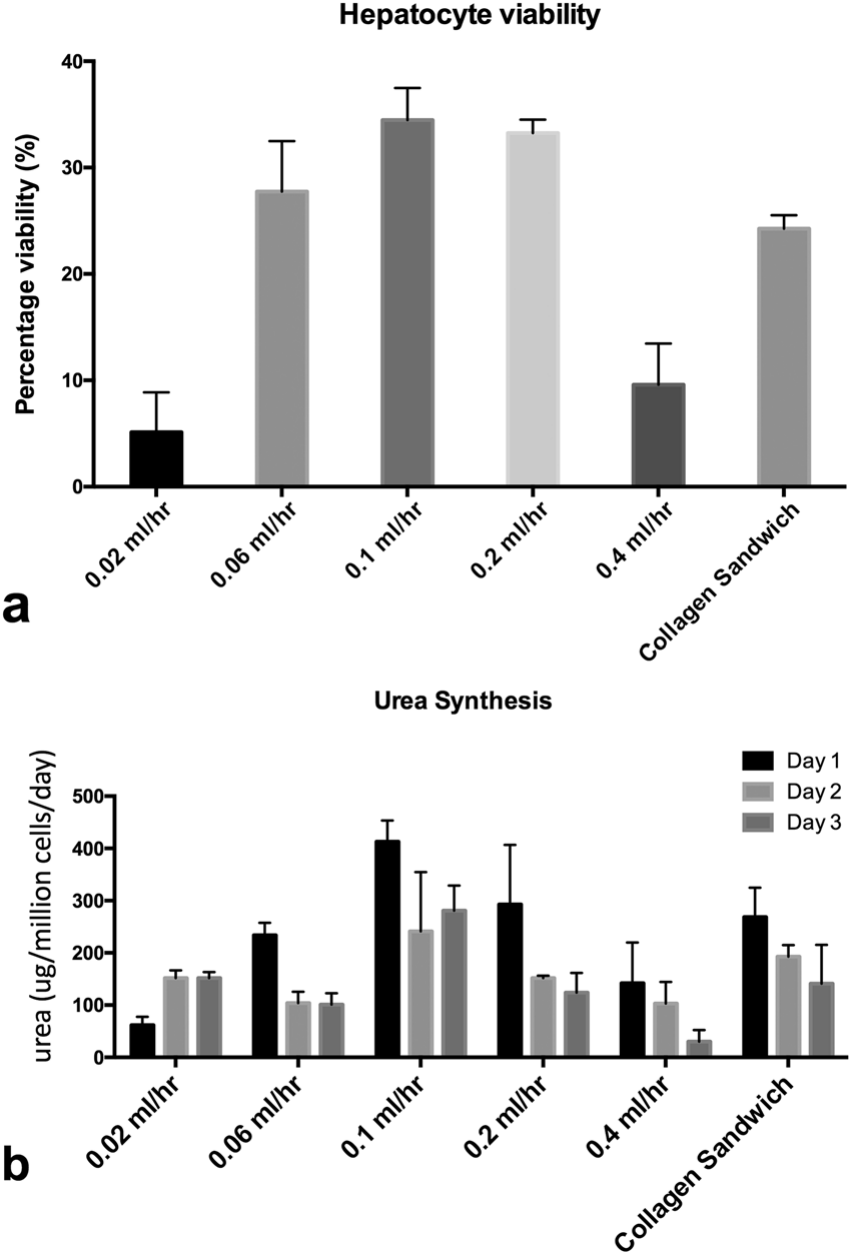
Optimization of the PIC system: (**a**) hepatocyte viability at different flow rates (**b**) urea production during 3 days culture for different flow rates. The working flow rate range is between 0.6 and 2 mL/h, the optimum point is 0.1 mL/h.

The decrease along the depth direction is less significant. This may be attributed to the shallowness of the cell culture chamber (H = 0.5 mm) in which the O_2_ transfer from the top to bottom is relatively fast. Figure 4a and b show the averaged O_2_ concentration and shear stress at the membrane plane. The simulation was performed for different depth of the bioreactor (0.5, 1, 2 and 3 mm). With the increasing flow rate, more O_2_ is carried into the system by the culture medium while the consumption rate is not significantly affected. As a result, the O_2_ concentration at the membrane level also increases, although the change is less significant when the flow rate is beyond 0.1 mL/h. On the other hand, for an increased depth of the cell culture chamber a lower O_2_ concentration (at the same flow rate) was achieved, which is not surprising due to the corresponding higher resistance for O_2_ transfer. Moreover, the shear stress on the membrane can increase almost linearly with the increasing flow rate, as shown in Fig. 4b. It can be observed from the slope of the lines that a shallow depth corresponds to a high shear stress. For example, at the same flow rate, the shear stress for H = 0.5 mm is about 38 times higher than that for H = 3 mm. A typical enigma in designing a cell culture chamber is to achieve a compromise between the requirement for mass transfer and the protection of cells from shear stress. A shallow chamber operating at high flow rates may provide better nutrient supply and waste removal than a deeper one at low flow rates. However, the former usually generates higher shear stress on the cells than the latter, as shown by the simulation results. In an environment with either insufficient nutrients and O_2_ or too much shear stress, the cells cannot exhibit proper metabolism or even become unviable. With the help from the porous membrane, used in this study to shield cells from most of the shear stress, it is possible to design a shallow chamber (e.g. H = 0.5 mm) and operate it at a relatively high flow rate (e.g. Q = 0.1 mL/h), in an effort to provide sufficient O_2_ to the cells for their metabolic activities. We will further demonstrate that the cell viability can be affected by the shear stress at high flow rates (Fig. 5a).

The O_2_ equilibrium is affected by not only the supply and transfer but also the consumption rate. Figure 4c shows the O_2_ level at the membrane plane with different number of cells cultured in the chamber. Obviously, a larger number of cells correspond to higher O_2_ consumption, resulting in a lower oxygen level in the region with cells.

#### Optimization of the flowing conditions

Flow rate was optimized with flow simulation and verified by selecting a flow rate at which mass transfer is maximized without too much shear stress damage to the cells to ensure the best performance of the on-chip culture. Hepatocyte spheroids were cultured under different flow rates: 0.02 mL/h, 0.06 mL/h, 0.1 mL/h, 0.2 mL/h and 0.4 mL/hr. MTS assay and urea assay is performed after 3 days of experiment to evaluate hepatocyte viability and function for different flow rates. To compare with static cultures, MTS activity of spheroids in PIC was measured in 48 well-plate, after transferring the coverslips from the PIC back to the well-plate. The results are presented in Fig. 5a (hepatocytes viability) and Fig. 5b (urea production). The spheroid viability in the chip and collagen sandwich were benchmarked against the cell viability of freshly isolated hepatocytes seeded in the 48 well-plate. Due to cell loss in the process of spheroid formation and transfer of coverslip, the viability presented are around 30–40% of control. The working range is between 0.06 and 0.2 mL/h, having an optimum flow rate at 0.1 mL/h.

#### Cell culture chamber depth affects cell viability and function

**Figure 6:**
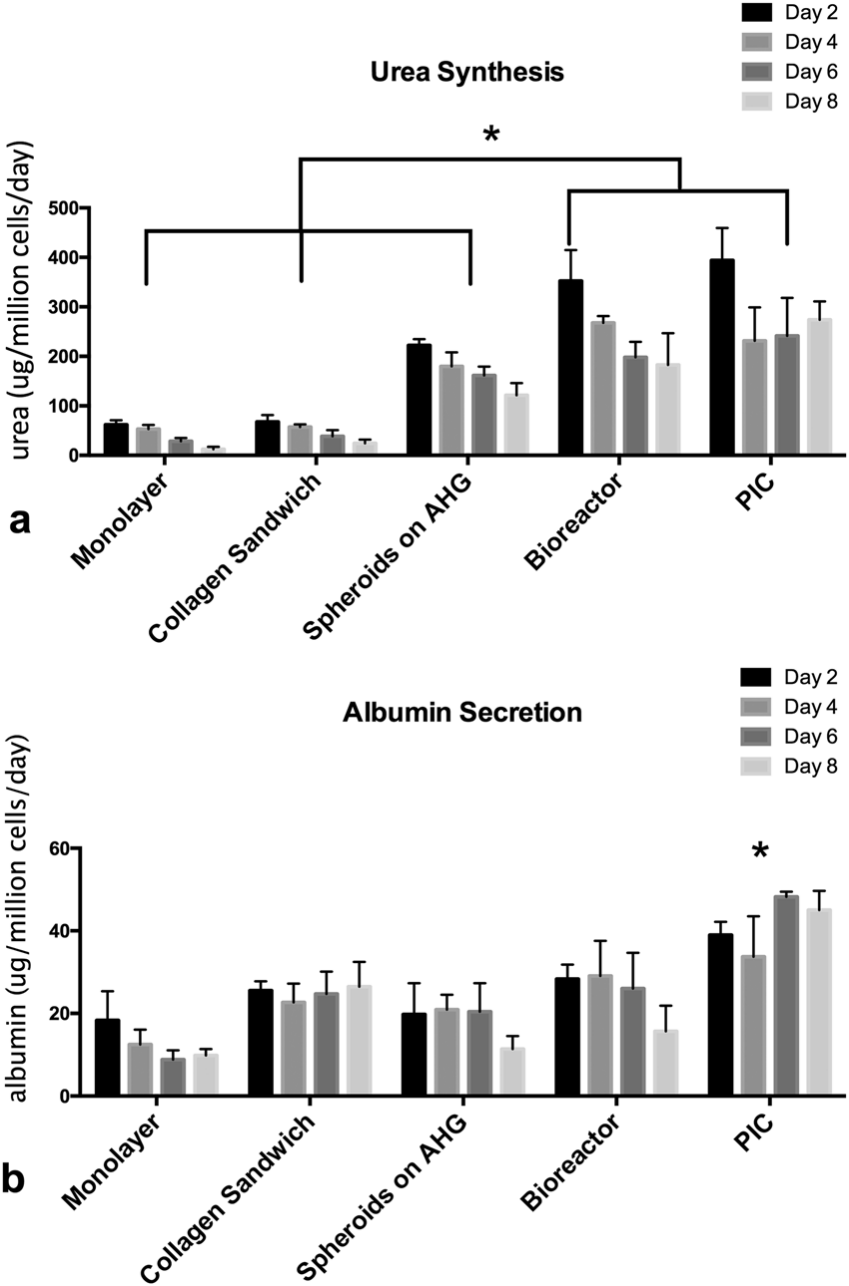
Comparison of cellular functions over 8 days in PIC and a laminar flow bioreactor. (**a**) The cells cultured in PIC and the bioreactor produced higher levels of urea compared with static cultures. (**b**) Albumin production in PIC is significantly higher than other groups. Results were obtained from triplicate measurements (n = 3), *p ≤ 0.05. Data from representative experiments are presented, whereas similar trends were seen in multiple trials.

The influence of chamber depth was studied. Theoretically, a tangential flow over the surface of the membrane helps the removal of the metabolites and by-products and refresh the media around the cells. Moreover, the concentration of the O_2_ and nutrients decrease with the depth of the cell culture chamber (Fig. 6a), so we expect an optimal hepatocyte function and viability can be identified at optimal depth. We experimented with different depth of the cell culture chamber: 0.5 mm, 1 mm, 2 mm and 3 mm in order to appreciate the relevance of this parameter. The experiments are presented in detail in Supplementary 3 the cells cultured in chamber with 0.5 mm depth showed the highest secretion level.

#### Experimental validation of oxygen concentration simulation results

At the flow rate of 0.1 mL/h, the calculated O_2_ consumed by the cells is 7.0%, 7.8%, 9.2% and 10.5% (from O_2_ level in the fresh media) for the depth of 0.5 mm, 1.0 mm, 2.0 mm and 3.0 mm, respectively (Fig. 4a). This trend similar to the experimental observations in Supplementary 3: cells cultured in thinner cell culture chambers had better cell viability and metabolic functions due to better supply of O_2_ and nutrients. We tested the drop in O_2_ concentration in culture media after cell culturing using fluorescence signal of platinum-octaethyl-porphyrin (PtOEP) dye (method described previously^25^). The O_2_ concentration can be quantified because of PtOEP’s oxygen-induced phosphorescence quenching effect. The results are shown in Supplementary 4 indicating a drop in O_2_ concentration of ~10%.

#### Experimental validation: temperature of the cell culture media in PIC

The temperature of the cell culture media was verified in the flowing conditions (0.02 to 0.4 mL/h) using a thermocouple. The thermocouple was placed (using a modified PDMS cover) inside the cell culture well as well as in the outlet microfluidic connector. There was no difference between the indication of the temperature controller and the measurements, showing good thermal transfer of the design system.

#### Absorption of drugs to the PIC

The accuracy of drug testing experiments performed in microfluidic devices depends on achieving a consistent and reproducible concentration of drug molecules in the solution. However, in microfluidic devices with PDMS components, hydrophobic molecules tend to be absorbed to the walls of microfluidic devices, which reduces their concentration and affect the accuracy of drug testing results. We quantified the absorption of drugs to the tubing and microfluidic chip, the results are presented in Supplementary 5. The surface area of the PDMS cover (~450 mm^2^) exposed to the media in the PIC was much smaller than the surface area in the tubing (~2300 mm^2^). Majority of the drug absorption was found to be attributed to the tubing. Initially, after 30 minutes of perfusion, 74% of the APAP can be retrieved from the outlet, only 52% of the diclofenac can be retrieved. After 48 hours of perfusion, drug molecules absorbed to the surface starts to saturate, 98% of APAP and 95% of diclofenac can be retrieved. Diclofenac absorbs more easily to PDMS surface than APAP because of its hydrophobicity and higher log P value. To avoid cross-contamination of the drugs due to absorption, the PDMS cover of the chip and the tubing were not reused in the experiment.

#### Comparison with a laminar flow bioreactor

To benchmark the performance of the PIC against other perfusion culture systems, we compared the functions of cells in a bioreactor reported previously^26^ and the PIC (in both cases we use the same constrained spheroids cell culture model). The functions are compared with the cells cultured in 3 other control configurations. Monolayer setting refers to seeding hepatocyte directly to cell culture plates. Spheroids on AHG has the same substrate modification method and cell-culture configuration as PIC but cultured in multi-well plates. In this setting, hepatocytes are seeded on a low-adhesion substrate (glass coverslips modified with poly(ethylene glycol) (PEG) and galactose ligand 1-O-(60-aminohexyl)-D-galactopyranoside (AHG, MW 279)) to promote spontaneous spheroid formation^20^. We previously demonstrated that hepatocyte spheroids cultured on AHG-modified substrate exhibited improved polarity and liver-specific functions^27^. Collagen sandwich configuration is the industry standards that has often been recommended in FDA guidelines for *in vitro* hepatotoxicity testing^28^.

We measured the urea synthesis and albumin secretion levels in both perfusion culture systems over 8 days (Fig. 6). The cells cultured in PIC and the bioreactor produced similar levels of urea while both higher than static culture as expected. The albumin production in PIC is significantly higher than the laminar flow bioreactor. These results indicate that tangential flow that characterize the PIC structure is essential in maintaining cell functions.

We used experiment success rate to gauge the robustness of PIC compared with bioreactor. All the 12 experiments conducted with PIC and collagen sandwich were successful without contamination. 9 out of 12 experiments with spheroids cultured on AHG were successful, with 3 experiments having extensive cell detachments after 7 days of culture. Only 3 out of 12 experiments were successful for laminar flow bioreactor, mostly because of bacterial contamination issue during the culture. All the experiments were conducted by the same operator.

### Maintenance of cell function in PIC

#### Long-term cell viability on chip

**Figure 7:**
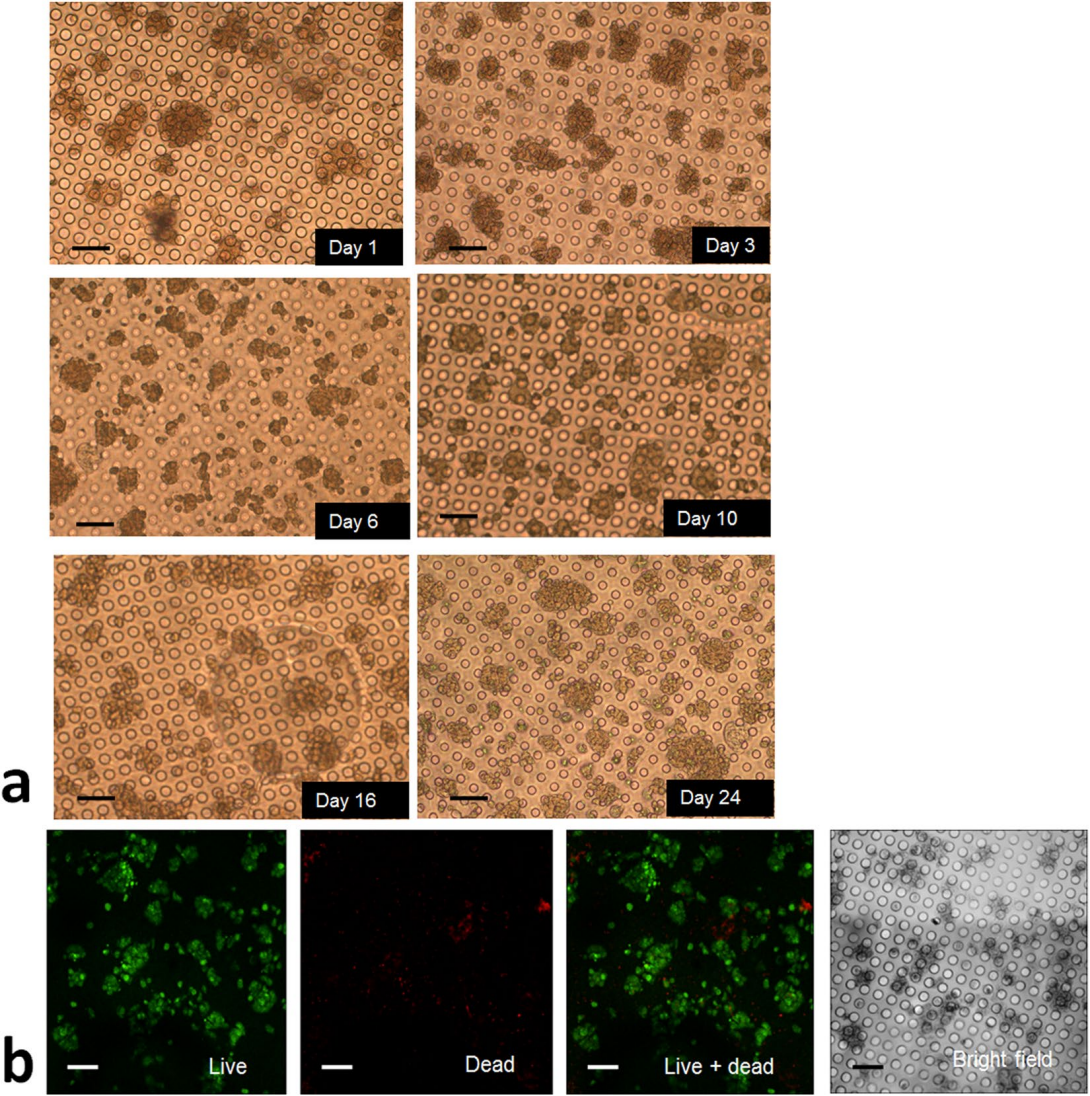
(**a**) Phase contrast images with constrained spheroids during 24 days cell culture. Cell viability can be maintained up to day 24; (**b**) live/dead assay at day 24, showing viable cells with intact spheroid structure. Scale bar represents 100 μm.

The PIC system for hepatocyte constrained spheroid culture has been tested for long-term cell culture for 24 days. Optical images taken during this period of the constrained spheroids are presented in Fig. 7a. At day 24 a live/dead staining was performed. The images presented in Fig. 7b demonstrates the viability of the cultured cells can be maintained for 24 days.

#### Maintenance of hepatocyte differentiated function

**Figure 8:**
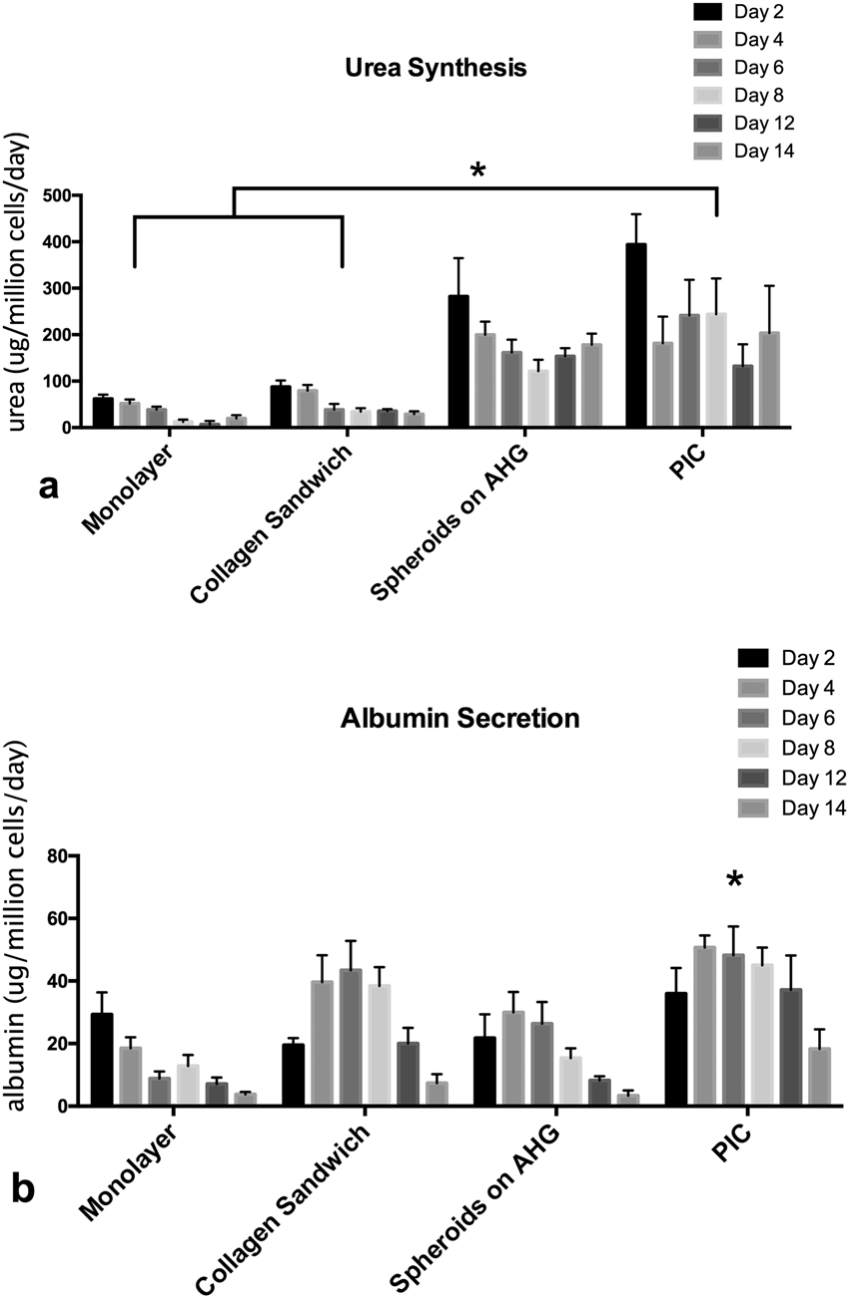
Synthetic function of hepatocytes cultured in monolayer, collagen sandwich, constrained spheroid in static culture and constrained spheroid on PIC over 14 days. (**a**) Hepatocytes cultured in PIC produced significantly higher amount of urea compared with monolayer and collagen sandwich. (**b**) Albumin secretion is significantly higher in PIC than other groups. Results were obtained from triplicate measurements (n = 3), *p ≤ 0.05. Data from representative experiments are presented, whereas similar trends were seen in multiple trials.

To investigate whether the PIC can support chronic hepatotoxicity testing of drugs, we studied whether the hepatocyte functions can be maintained over 14 days. The results of urea secretion and albumin synthesis are presented in Fig. 8a and b respectively.

Urea synthesis in spheroids on AHG increased compared to collagen sandwich culture (Fig. 8a); the increase was more significant than the increase in albumin secretion (Fig. 8b). The urea produced by hepatocytes cultured in collagen sandwich configuration is 87.7, 79.4, 38.4, 34.5, 35.5, 28.9 µg/10^6^ cells/day on day 2, 4, 6, 8, 12 and 14 respectively. By comparison, the urea secreted by spheroids on AHG ranged from 282.3, 199.9, 161.6, 121.6, 153.6, 178.3 µg/10^6^ cells/day on day 2, 4, 6, 8, 12 and 14 respectively. Urea secretion further improved in PIC: 393.7, 181.5, 241.5, 243.76, 132.5, 203.4 µg/10^6^ cells/day on day 2, 4, 6, 8, 12 and 14 respectively. Notably, hepatocytes cultured in PIC secreted about 7 times the amount of urea compared with collagen sandwich.

Hepatocytes cultured in PIC showed significant increase in albumin secretion compared to collagen sandwich throughout 14 days of culture (Fig. 8b). The amount of albumin secreted by collagen sandwich cultured hepatocytes on day 2, 4, 6, 8, 12 and 14 were 19.5, 39.6, 43.4, 38.4, 20, 7.3 µg/10^6^ cells/day respectively. The amount of albumin secreted by spheroids on AHG in the same period were 21.8, 29.9, 26.4, 15.3, 8.2 and 3.3 µg/10^6^ cells/day on day 2, 4, 6, 8, 12 and 14 respectively. Albumin secretion capacity of spheroids on AHG improved in perfusion culture. The amount of albumin secreted by hepatocytes in PIC on day 2, 4, 6, 8, 10, 12 and 14 were 35.9, 50.7, 48.2, 45, 37.17, 18.3 µg/10^6^ cells/day respectively.

We quantified the gene expression levels of CYP1A2, CYP2B1/2 and CYP3A2 in PIC and compared with monolayer and collagen sandwich culture. These CYPs are rat homologs of human CYP1A2, CYP2B6, and CYP3A4 respectively. They have important roles in drug metabolism. CYP1A2 is the second most abundant CYPs in human body and is inducible by many of the carcinogens and aromatic hydrocarbons. CYP2B6, although only account for 5% of total CYP component, is responsible for the metabolism of more than 25% of drugs in the market. CYP3A4 is the most abundant in human liver and responsible for metabolism of two-thirds of all marketed drugs.

**Figure 9:**
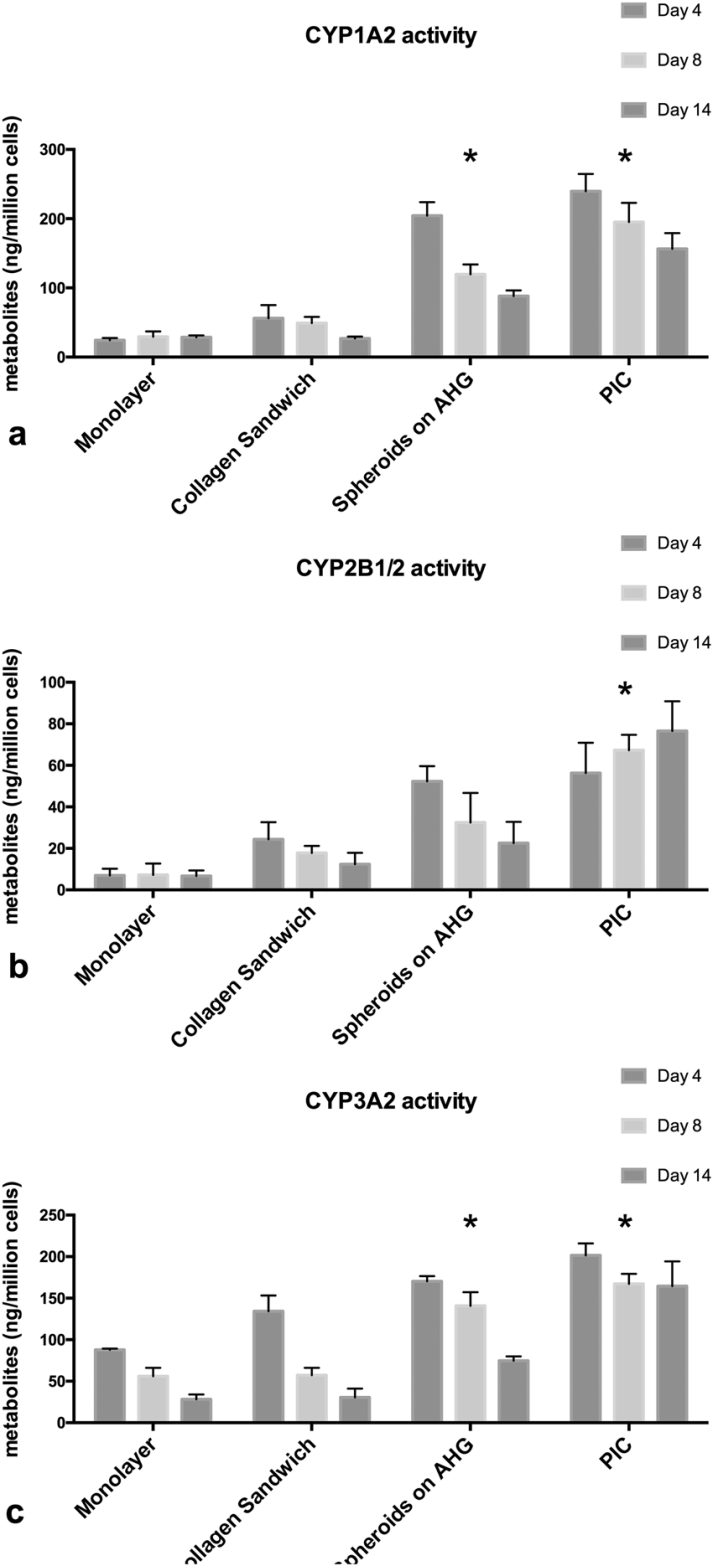
Higher CYPs enzymatic activity for PIC cultured hepatocytes compared with monolayer, collagen sandwich and Spheroids on AHG. (**a**) CYP1A2 activity, measured by the amount of acetaminophen metabolized from phenacetine; (**b**) CYP2B1/2 activity, measured by the amount of OH-bupropion metabolized from bupropion; (**c**) CYP3A2 activity, measured by the amount of 1′-OH-midazolam metabolized from midazolam. Results were obtained from triplicate measurements (n = 3), *p ≤ 0.05. Data from representative experiments are presented, whereas similar trends were seen in multiple trials.

The activities of CYP1A2, CYP2B1/2 and CYP3A2 enzymes were measured using their respective specific substrates: phenacetine, bupropion and midazolam (Fig. 9a). The quantity of their respective metabolite acetaminophen, OH-bupropion and 1′-OH-midazolam were measured with LC-MS. We found that hepatocytes cultured in spheroid model shows enhanced CYP1A2 activity; the amount of acetaminophen (metabolite of phenacetine metabolized by CYP1A2) produced by collagen sandwich cultured hepatocytes was measured at 49.2ng/10^6^ cells/90 min, while hepatocytes in PIC produced 195.1ng/10^6^ cells/90 min on Day 8.

On day 14, the amount of acetaminophen measured in collagen sandwich and PIC were 26.9ng/10^6^ cells/90 min and 156.4ng/10^6^ cells/90 min respectively.

The CYP2B1/2 activity of hepatocytes was quantified by measuring the amount of OH-bupropion metabolized from bupropion (Fig. 9b). On day 8, the amount of OH-bupropion produced by collagen sandwich was 17.78 ng/ 10^6^ cells/90 min, lower than spheroids on AHG, which produced 32.49 ng/10^6^ cells/90 min of OH-bupropion. By comparison, Hepatocytes in PIC produced significantly higher OH-bupropion: 67.32 ng/10^6^ cells/90 min on day 8. On day 14, the amount of OH-bupropion produced by collagen sandwich and spheroids on AHG were 12.38 ng/10^6^cells/90 min and 22.54 ng/10^6^cells/90 min respectively. The amount of OH-bupropion produced by PIC cultured hepatocytes was slightly higher at 76.56 ng/10^6^cells/90 min but this is not statistically significant.

To quantify the activity of CYP3A2, we measured the amount of 1′OH-midazolam metabolized by CYP3A2 from midazolam (Fig. 9c). On day 8, the amount of 1′-OH-midazolam produced by collagen sandwich and spheroids on AHG were 57.2ng/10^6^ cells/90 min and 140.6 ng/10^6^ cells/90 min respectively. The amount of 1′- OH midazolam produced by spheroids in PIC was higher at 167.2ng/10^6^ cells/90 min. On day 14, collagen sandwich and spheroids on AHG produced 30.6ng/10^6^ cell/90 min and 74.6ng/10^6^ cell/90 min of 1′-OH-midazolam. By comparison, spheroids in PIC produced 164.5 ng/10^6^ cells/90 min of 1′-OH-midazolam.

#### Application of PIC-cultured hepatocytes in drug safety testing

We studied the hepatocyte spheroids under static and perfusion (PIC) conditions for two weeks for the evaluation of chronic drug effects. Acute toxicity and chronic toxicity of two drugs: Diclofenac and Acetaminophen (APAP) were tested and compared. Diclofenac causes rare but significant cases of serious hepatotoxicity including liver necrosis, jaundice, fulminant hepatitis with and without jaundice, and liver failure^29^. Diclofenac toxicity is difficult to be evaluated due to the problematic estimation of the dose response. Its hepatotoxic effects are not easily reproducible in current animal models, indicating idiosyncratic toxicity of diclofenac^29^. Therefore, the liver function should be monitored on long-term therapy with diclofenac since increased AST/ALT levels are observed in clinical treatment^30^. *In vivo*, delayed diclofenac induced hepatotoxicity usually occurs^29^. Previous studies showed that *in vitro*, only high concentrations of diclofenac (>200 µM) induce acute toxicity^30,31^ in primary cultures of human hepatocytes. Thus diclofenac repeatedly dosed over 2 weeks in our *in vitro* PIC was tested. For APAP as a most frequently used pain killer in the world, there is large body of literature characterizing its safety *in vitro* and *in vivo* ^32–34^. Both acute and chronic overdosing of APAP can cause liver toxicity and liver failure^32^.

**Figure 10:**
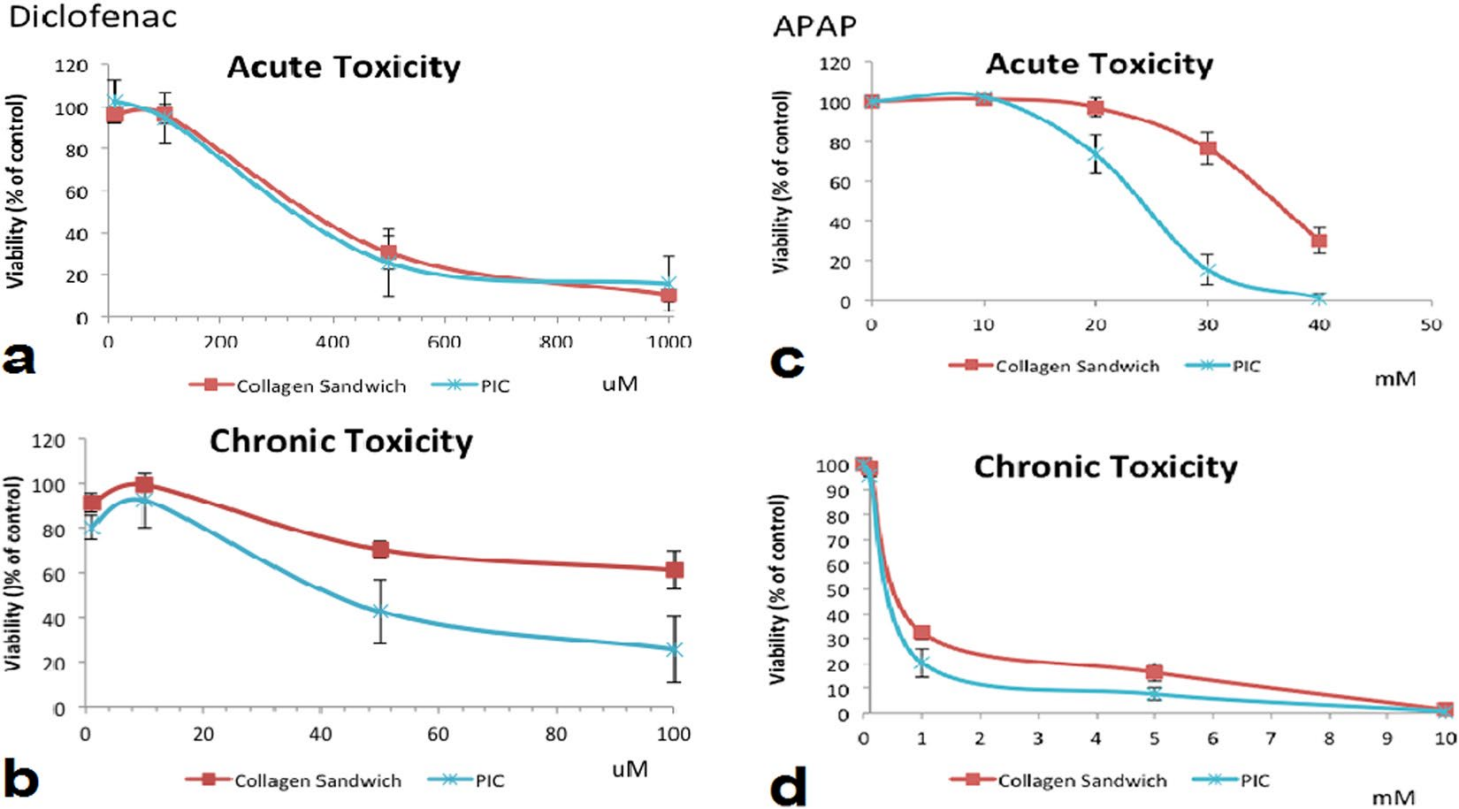
Comparison of acute and chronic dose response to (**a,b**) Diclofenac and (**c,d**) APAP between collagen sandwich and PIC (day 1 and day 14), Quantified MTS levels were normalized to respective control conditions. Results were obtained from triplicate measurements (n = 3). Data from representative experiments are presented, whereas similar trends were seen in multiple trials.

Acute toxicity (24 h) of diclofenac is presented in Fig. 10a. In this experiment, we observed a significant toxic effect at 500 µM and 1 mM concentration. At the highest concentration (1 mM), the cell viability significantly decreased to 10%. There is no relevant difference between PIC and collagen sandwich culture for diclofenac acute toxicity. The IC50 was calculated to be 495.61 µM and 493.66 µM respectively. For APAP acute toxicity, we observed that cells cultured in PIC were more sensitive to APAP treatment than the cells cultured in collagen sandwich (Fig. 10c). The IC50 of APAP was 22.51 mM and 38.57 mM for PIC and collagen sandwich.

Chronic drug toxicity of diclofenac and APAP were evaluated by measuring the viability of hepatocyte spheroids perfused with different drug concentrations for 14 days. For chronic hepatotoxicity, static cultured hepatocytes (Fig. 10b), the lowest cell viability was found at 100 µM diclofenac. In the perfusion culture, spheroids were exposed to repeated dosing of diclofenac every 2 days. The perfusion system is more sensitive for testing chronic drug response of diclofenac: the IC 50 is 50.86 µM for PIC and 121.53 µM for the collagen sandwich control. At 100 µM diclofenac chronic dosage, cell viability is reduced to less than 40% in PIC after 14 days of culture, whereas 100 µM diclofenac dosing doesn’t show a significant acute toxicity. Similarly, cells cultured in PIC is more sensitive to chronic APAP toxicity (Fig. 10d): at 1 mM concentration, cell viability was reduced to 21% in PIC, whereas cells cultured in the collagen sandwich control had 32% viability compared to the DMSO control. The IC 50 is 2.391 mM for PIC and 3.059 mM for the collagen sandwich control.

## Discussion

*In vitro* liver cell models based on conventional cell culture platforms such as 2D monolayer and collagen sandwich can support hepatocyte function for a week. 3D cell culture configurations can enhance functions to higher level with their own limitations such as mass transfer and heterogeneous environments etc. Bell *et al*. proposed a 3D primary human hepatocyte model for *in vitro* study of liver functions and diseases and drug response^35^ Perfusion culture models have been developed to regulate the soluble microenvironment and can sustain hepatocyte function in a confined space for a longer period, such as suspension spheroids perfusion-stirred bioreactor^36^, encapsulated spheroids bioreactor^37^ and laminar flow spheroids bioreactor^27^. Various microfluidic chips have been developed to provide physiological shear stress and liquid to cell ratio within the microfluidic compartments for a few days^38–41^. Carraro *et al*.^42^ proposed a perfusion hepatocyte cell culture having a “vascular-like” microfluidic structure in hepatocyte culture seeded on a nanoporous membrane. Vernetti *et al*.^43^ proposed a 3D human liver model organized in four-cell sandwich structure (hepatocytes, endothelial cells, stellate cells and Kupffer cells). Lee *et al*.^44^ fabricated a PDMS device which mimics the liver anatomy. The device features an artificial liver sinusoid with an artificial barrier layer mimicking endothelial barrier layer. We have also integrated cell lines and primary cells into 3D microfluidic channels to evaluate the cellular response to drug exposure^4,26,27,45–47^. However, these devices have not been utilized in chronic drug safety testing applications due to the deterioration of hepatocyte functions and contamination problems associated with repeating dosing of drugs to the cells. A number of chips have been developed for long-term culture^48–52^. Wagner *et al*.^53^ combined traditional PDMS microfluidic device with transwell inserts and created a multi-organ chip capable of supporting long-term cell culture. Maschmeyer and colleagues^54^ developed a PDMS multi-chamber chip for long-term co-culture of 4 tissue types. Recently, Kang *et al*.^49^ presented a long-term transwell microfluidic device using PDMS microchannels and polyethyleneterephthalate (PET) membranes to mimic the liver sinusoid structure. These devices have the advantage of adjusting fluid flow and controllable liquid to sample ratio, enabling prolonged cell culture. However, these PDMS devices all rely on traditional incubator and involve complex fluidic connections to operate. The drawback of these devices is that when they undergo frequent transfer of devices from incubator to microscope for observation, changing media and adding drug doses, the risk of contamination and bubble generation- once the chip is reinserted in the microfluidic setup- becomes higher. Our PIC is such a device that integrates incubator functions such as temperature and pH control, and with active debubbler for 3D long-term culture and systematically ensure contamination-free long-term cell culture for repeatedly dosed drug testing. This PIC system doesn’t need to be inserted in an incubator system, it works independently in a biosafety cabinet. It enabled long-term maintenance of 3D hepatocyte spheroid culture. We tested cell viability for 24 days, while the cell function was verified for 2 weeks. The integration of all the elements such as temperature controller and active debubbler allowed constrained spheroid model to be cultured in a stable microenvironment for weeks. Heaters have been used in cell culture platforms adapted to robotic systems and real-time imaging systems^55–58^. We applied this concept to our long-term cell-culture chip and implemented a thermocouple and a temperature controller to achieve precise maintenance of temperature during prolonged culture period. We have created a closed microenvironment for cell culture and manipulation inside a sterile biosafety cabinet, eliminating the need of an incubator. Moreover, the tangential flow allowed refreshing of media, removing of by-products and protection of the spheroids cell culture from shear stress.

Microfluidic cell-culture systems typically need to be pre-conditioned and sterilized before cell seeding^59^. Bubbles can form at fluidic connections or generated from the dissociated gas in the media. A great deal of care is needed during the operation of microfluidic chips to ensure bubble-free condition in the cell culture. In the long-term cell-culture chip, the chance of bubble accumulation in the microfluidic channels is even higher, amplifying the effects of bubbles to perfusion flow and cell function. Bubble traps^60–62^ are usually used to prevent bubbles from entering cell culture area of the microfluidic chips. When the bubble trap is filled with bubbles over weeks, additional bubbles can still enter cell culture areas. Therefore, we integrated a bubble trap and an active-debubbler to trap and remove the bubbles generated during long-term culture.

A long-term cell-culture system must also maintain a microenvironment with favourable mass transport and limited shear stress. In this study, we used an established hepatocyte culture model: the constrained spheroid model, due to its superior ability to maintain cell function and minimize cell loss^27^. Here, we further optimized the spheroid seeding and perfusion culture process. We pre-formed the spheroids on glass coverslips and constrained them with parylene C ultra-thin porous membrane in 48-well plates. After spheroid stabilization, they are transferred to the PIC chip to take advantage of the perfusion flow. We demonstrated improved and well-maintained activity of hepatocytes’ CYP1A2, CYP2B1/2 and CYP3A2 enzymes. We validated the use of PIC for acute and chronic drug toxicity studies. We found that for APAP, the acute toxicity response is more sensitive in the PIC compared to static culture. Similar APAP toxicity testing results^63^ were reported in perfusion bioreactor: perfusion of APAP resulted in a shift in dose response such that 20 mM of APAP in perfusion culture lead to significant acute toxicity compared with 40 mM in static culture. The reason is that, the metabolic activity is elevated in perfusion culture microenvironment. Hepatocytes can metabolize APAP faster and produce higher quantity of its reactive intermediate NAPQI, which causes free-radical damage of cellular structures^64^. We further validated the use of our PIC chip for chronic drug testing by evaluating hepatotoxicity after 2 weeks’ exposure to diclofenac and APAP and obtained a more sensitive chronic toxicity response in the perfusion-cultured spheroid model compared with the static cultured collagen sandwich control. Chronic toxicity of diclofenac is more sensitive in the PIC compared with static culture. Diclofenac chronic toxicity is largely attributed to one of its phase II metabolite: diclofenac-acylglucuronide due to covalent binding^65^. Phase II metabolism requires longer time for metabolite production than primary metabolites. Therefore at 100 mM concentration, diclofenac doesn’t show acute toxicity but results in chronic toxicity.

The PIC is currently a low throughput chip that addressed the contamination issues associated with long-term culture of cells in 3D to preserve high levels of cell function for drug testing applications. In the near future, such *in vitro* model could be used to study the complex spatiotemporal cues that govern long-term changes in cell fates. The PIC can be further developed into high-throughput format, to involve multiple cell types in co-culture, and to support industry-scale drug screening applications.

In conclusion, we presented a method and a system for robust long-term hepatocytes constrained spheroid culture for chronic drug testing. The PIC integrates a heater and temperature controller to control temperature, CO_2_ pressure-driven culture-media flow to control pH, and active debubbler on chip, in a biosafety cabinet, to minimize the risk of contamination. The tangential flow over the parylene membrane (that stabilize the spheroids and limit the shear stress) assures a controlled microenvironment. The PIC maintained the cell functions for 2–3 weeks, supporting repeated dosing chronic drug testing. This PIC is a robust long-term 3D perfusion culture platform that can be used also for culturing different cell types for other sub-acute and chronic drug testing applications.

## Methods

### Flow simulation

As a powerful tool based on the transport theories of momentum, mass and energy, computational fluid dynamics (CFD) simulation yields the profiles of shear force and O_2_ concentration in this study. The geometry and mesh of the flow domain were constructed using GAMBIT 2.3.16, and the created mesh was introduced into FLUENT 6.2.16 for calculation. The flow was considered as laminar, steady and three-dimensional, with the operating temperature of 37 °C. The inlet and outlet of the bioreactor were modelled as “velocity-inlet” and “outflow”, respectively, while the walls were considered as no-slip. In addition to the Navier–Stokes equations, a species transport equation was solved to calculate the oxygen concentration in the bioreactor. The concentration of dissolved O_2_ at the inlet was set as 6.08e-3 kg/m^3^ according to its solubility in plasma (*α*) at 37 °C. The zone occupied by the hepatocytes under the membrane was modelled as a “source term” for O_2_, from which the dissolved oxygen was consumed at the Oxygen Uptake Rate (OUR) of the hepatocytes^66^.

1 $$OUR={V}_{m}\times \frac{c/\alpha }{c/\alpha +{K}_{m}}$$

where *c* is the local O_2_ concentration; *V*_m_ is the maximum rat hepatocytes O_2_ consumption rate; and *K*_m_ is the half-saturation constant for hepatocytes. The parameters were taken from Table 1 of the reference^66^. Since *c* is an unknown variable in Eq. (1), a user-defined-function (UDF) was used to determine the O_2_ source in an iteration manner. A simple calculation shows that OUR decreases by only 3% when *c* decreases by 50% from its solubility, indicating that the O_2_ consummation rate is not very sensitive to the change of local O_2_ concentration in this range. The number of hepatocytes, unless otherwise specified, was set at 23,200, identical to that used in experiments. The calculation process was continued till all the residues for continuity, momentum and species fell below 10^−7^.

### Rat hepatocytes isolation and spheroid pre-culture

We used a constrained spheroid model established previously^27^ as the hepatocyte cell culture model. Hepatocytes aggregated in spheroids are able to retain polarity and form functional bile canaliculi^67^. Functional parameters such as albumin and urea production, CYP450 activities are also improved compared with collagen monolayer^19,68^ and collagen sandwich culture^69,70^.

To obtain hepatocyte spheroids, we isolated primary rat hepatocytes from male Wistar rats of 250–300 g in weight using a two-step collagenase perfusion method^37^. Animals were handled according to the Institutional Animal Care and Use Committee (IACUC) protocol. Isolation protocol was reviewed and approved by the Biological Resource Center (BRC) Institutional Animal Care and Use Committee. Freshly isolated hepatocytes were seeded onto collagen-coated 48-well plate or PEG-galactose modified glass coverslips at 1 × 10^5^ cells/cm^2^ to pre-form the spheroids^27^. The glass coverslips were modified according to the protocol^27^. The cells were cultured in William’s E medium supplemented with 1 mg/mL BSA, 10 ng/mL EGF, 0.5 μg/mL insulin, 5 nM dexamethasone, 50 ng/mL linoleic acid, 100 units/mL penicillin and 100 μg/mL streptomycin; and incubated with 37 °C, 5% CO_2_, 95% humidity. Cell seeding glass coverslips 10mm in diameter were purchased from Paul Marienfeld GmbH & Co.KG (Lauda-Königshofen, Germany). Silane-PEG-COOH, MW 5000 was purchased from Nanocs Inc. (New York, USA). 1-O-(6′-aminohexyl)-D-galactopyranoside (AHG, M.W. 279), the galactose ligand was synthesized in house as reported previously and verified by NMR spectrum^20,71^. Wafers used for chip fabrication were purchased from Bonda Technology (Singapore). Other chemicals were purchased from Sigma-Aldrich (Singapore) unless otherwise stated. Parylene C membranes used for sandwich constrained spheroid culture were fabricated and surface modified^27^. After 1 day in static culture, the coverslips with attached spheroids were manually moved to PIC using sharp point forceps and covered with parylene C membrane. The PIC was sealed by clamping the PDMS-glass cover on top of the culture chamber. To evaluate the robustness of the PIC, we conducted cell culture experiments in cell culture plates, in a laminar flow bioreactor previously developed^26^ and in the PIC in parallel. Six independent 14-day cell culture experiments were performed in duplicates for each setup.

### Collagen sandwich culture

We used both the hepatocyte monolayer and the collagen sandwich as standard controls for hepatotoxicity testing of drugs, in order to compare with the results achieved from the PIC. The bottom collagen-coating substrate was prepared by adding 40 µL neutralized collagen type I solution (Invitrogen, Palo Alto, USA) onto the 10mm glass coverslip before incubation at 37 °C overnight for gelation. Hepatocytes were seeded on the collagen-coated coverslip at 1 × 10^5^ cells/cm^2^ density; they were incubated for 1 h for full attachment before media replenishment and then cultured for 24 h. The culture medium was removed; subsequently, 40 µL of un-gelled collagen was overlaid on top of the cells. Gelation of the collagen overlay was allowed to occur at 37 °C for 3 h before fresh medium was replenished. The collagen sandwich culture was placed in the incubator with daily change of culture media.

### Urea and albumin synthesis measurement

1 mL of culture media were collected for urea and albumin synthesis measurements each day. Urea content in the culture media was measured using Urea Nitrogen Kit (Stanbio Laboratory, USA). Albumin concentration was measured using Rat Albumin ELISA Quantification Kit (Bethyl Laboratories Inc., USA). The absolute urea and albumin amounts were calculated based on media volume collected and was normalized against the cell number by the end of culture.

### Diclofenac and APAP concentration measurement

To determine the amount of diclofenac and APAP absorbed to the PIC and tubing, concentration of diclofenac^72^ and APAP^73^ were measured by colorimetric method using a Tecan infinite m200 plate reader (Tecan, Switzerland).

### Cell viability staining

The viability of hepatocytes was visualized by a dual staining method. 2 fluorescent nuclear dyes: Propidium iodide (PI) (Molecular Probes, USA) and Calcein AM (Molecular Probes, USA) were used to stain the necrotic and viable cell population. The staining was done by moving the glass coverslip from the PIC to a microscope slide. The cells were stained by incubating in 100 μl of 25 mg/mL PI (30 minutes) and 1 μM Calcein AM (30 minutes); the samples were washed 3 times with 1X PBS before imaging. All stained samples were imaged with a confocal microscope at 488 nm and 543 nm excitation.

### Liquid chromatography-mass spectrometry (LC-MS) measurement

For CYPs specific activity analysis, hepatocytes were cultured for 8 or 14days before adding CYP-specific substrates diluted in Krebs-Henseleit buffer (KHB) and incubated for 1.5 hours. Hepatocytes in perfusion cultured were removed from the bioreactor and transferred to multi-well plates for this experiment. The samples were collected and stored at −80 °C until LC-MS measurement. To conduct the LC-MS measurement, 50 µL of 100ng/mL internal standards were added to the samples and the mixture was dried using Techne® Sample Concentrator (Techne, UK). The dried residues were reconstituted using 100 µL of methanol containing 0.1% formic acid. The supernatant was then analysed using LC-MS system (LC: 1100 series, Agilent, US; MS: LCQ Deca XP Max, Thermo Finnigan, US) with 100 × 3.0 mm onyx-monolithic C18 column (Phenomenax, USA) as reported previously^74^.

### Acute toxicity

The hepatocyte spheroids were tested for acute toxicity. 1 day after culturing in PIC, The cells were exposed to 4 concentrations of diclofenac (10–1000 μM) and APAP (10–40 mM) for 24 hours under perfusion condition. DMSO vehicle controls were established by using medium supplemented with 1% DMSO without drugs. Viability was tested after removing the glass slides containing cells from PIC to a 48-well-plate. The viability was assessed with MTS assay (Promega, USA).

### Chronic toxicity

For the assessment of chronic toxicity of diclofenac and APAP, the hepatocytes were exposed to four concentrations of diclofenac (1–100 μM) and APAP (0.1–10 mM). The selection of these concentrations range corresponds to the therapeutic serum concentration (C_max_) of diclofenac (6.4 μM)^75^ and APAP (1 mM)^76^. Vehicle control (0.1% DMSO) was used. Serum free William’s E medium was used during drug treatment. Repeated doses were given every 48 h for static culture upon medium change. The viability was assessed with MTS assay (Promega, USA) upon 14 days after seeding of the hepatocytes.

### Statistical analysis

At least 3 experimental replicates were used for each data point. In each experiment, 2 chips were used to generate duplicated data. Statistical comparisons were performed using 2 way ANOVA. Results are expressed as means ± standard error of the means (sem) of 3 independent experiments.

## Electronic supplementary material

Supplementary information
