# Supplementary material for: A perfusion incubator liver chip for 3D cell culture with application on chronic hepatotoxicity testing
Source: Sci Rep. 2017 Nov 6;7:14528. doi: 10.1038/s41598-017-13848-5 (PMC5673965; doi:10.1038/s41598-017-13848-5)
Supplement: Supplementary file 1 — Supplementary information [file 41598_2017_13848_MOESM1_ESM.pdf]

A perfusion incubator liver chip for 3D cell culture with application on chronic hepatotoxicity testing

Supplementary Information

Fang Yu[1,2], Rensheng Deng[1], Wen Hao Tong[1,2], Li Huan[1], Ng Chan Way[2], Anik Islam Badhan[1], Ciprian Iliescu[1,7-9*], and Hanry Yu[1-6*]

[1]Institute of Bioengineering and Nanotechnology, A*STAR, The Nanos, 04-01, 31 Biopolis Way, Singapore, 138669, Singapore.

[2]NUS Graduate School for Integrative Sciences and Engineering, Centre for Life Sciences (CeLS), 28 Medical Drive, Singapore, 117456, Singapore

[3]MechanoBiology Institute, National University of Singapore, T-Lab, 5A Engineering Drive 1, Singapore, 117411, Singapore

[4]Department of Physiology, National University of Singapore, MD9 03-03, 2 Medical Drive, Singapore, 117597, Singapore, E-mail: nmiyuh@nus.edu.sg

[5]Singapore-MIT Alliance for Research and Technology, 1 CREATE Way, #10-01 CREATE Tower, Singapore, 138602, Singapore

[6]MechanoBiology Institute, National University of Singapore, T-Lab, 5A Engineering Drive 1, Singapore, 117411, Singapore

[7]National Institute for Research and Development in Microtechnologies, IMT-Bucharest, Bucharest 077190, Romania: E-mail: ciprian.iliescu@imt.ro

[8]Academy of Romanian Scientists, Splaiul Independentei nr. 54, sector 5, Bucharest 050094, Romania

BIGHEART, National University of Singapore, ND6, 14 Medical Drive, #14-01, Singapore 117599, e-mail: bigci@nus.edu.sg

* Corresponding authors

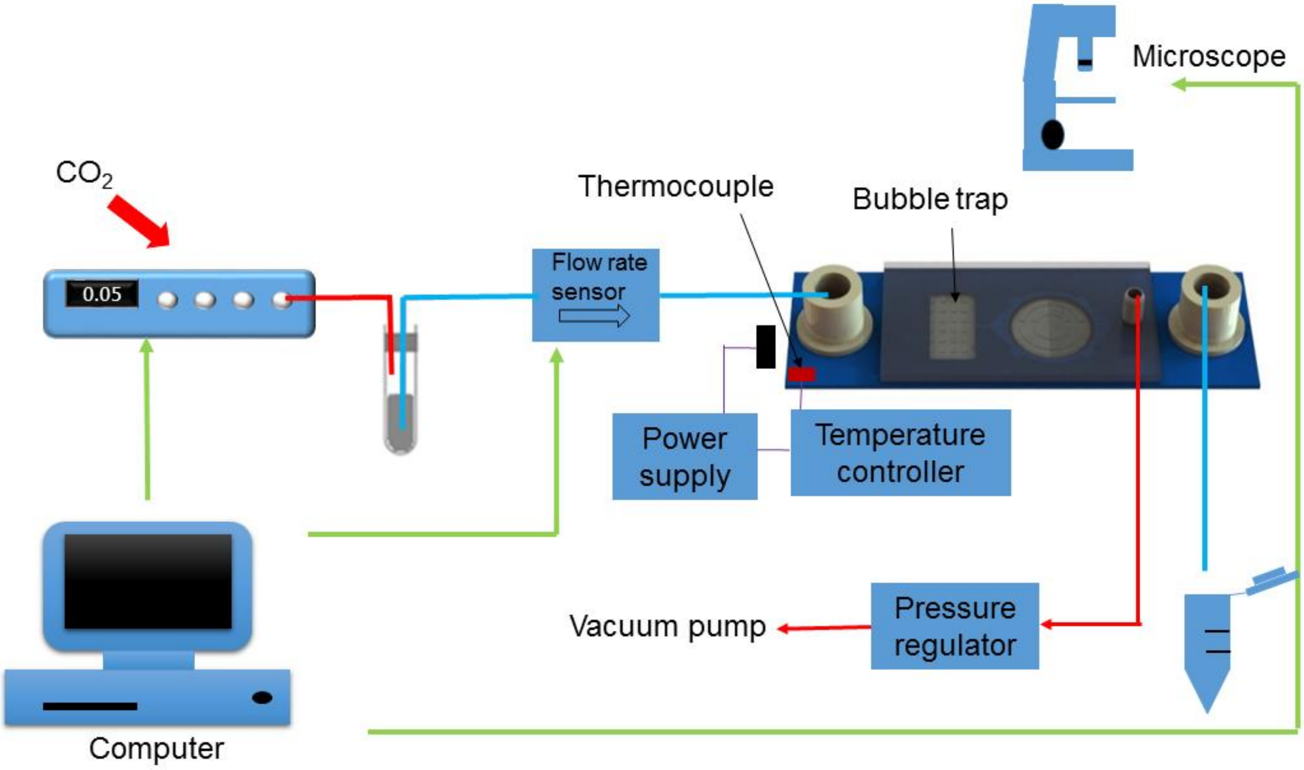

Figure S1: overall schematics of the microfluidic system

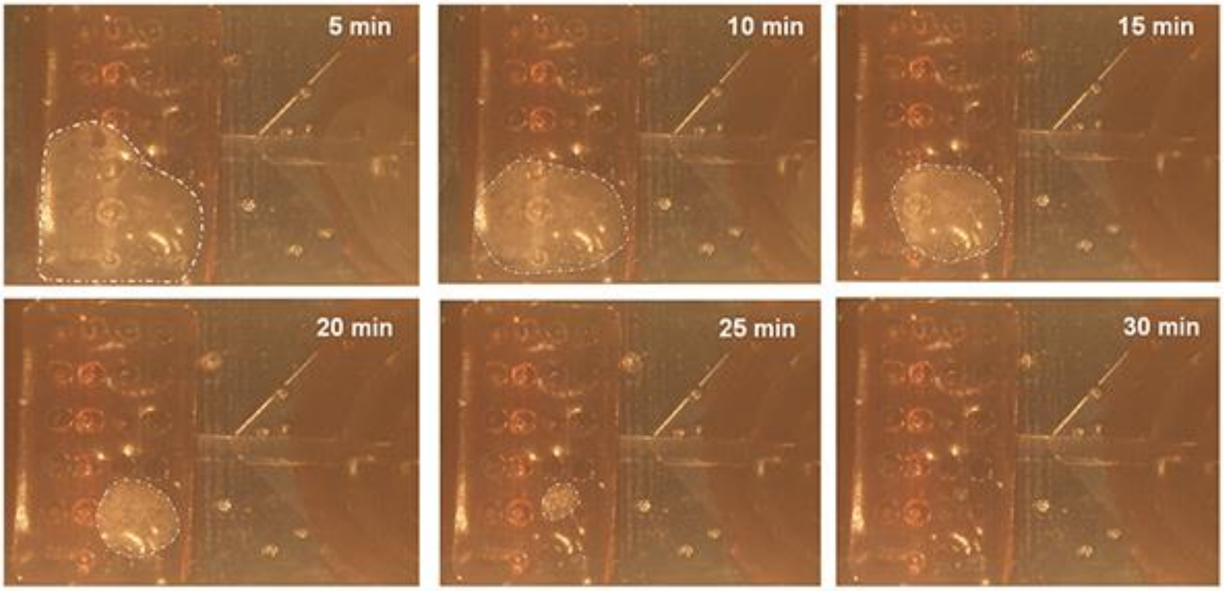

Figure S2: removal of the air bubble (shown in the dotted line) in the bubble trap. The bubble is completely removed after 30 minutes.

Supplementary 3: Influence of the cell culture chamber's depth:

Different depth of the cell culture chamber: 0.5mm, 1mm, 2mm and 3mm were fabricated in order to appreciate the relevance of this parameter The devices having 0.5 and 1mm chamber depth were fabricated as described in previous section. The chips having 2mm and 3mm chamber depth were fabricated assembling one or two silicon "spacers" -1mm-thick (with the corresponding etch-through holes). The assembly was performed using a thin PDMS layer using a contact imprinting method [73]. The experiments were performed for 3 days at a constant flow rate of 0.1mL/h. Cell viability (Fig. S3b) is not affected, but for albumin secretion (Fig. S3c) and urea synthesis (Fig. S3d), the cells cultured in chamber with 0.5mm depth showed the highest secretion level. Fig. 4a with Fig. 5a and Fig. S3b, show that the O_2 level increases sharply from Q=0.02mL/h to Q=0.06mL/h, and then increases moderately to Q=0.1mL/h. Correspondingly, cell viability increases sharply from Q= 0.02mL/h to Q = 0.06mL/h; and then moderately to Q =0.1mL/h before it decreases at Q=0.2 and 0.4mL/h due to excessive shear stress. On the other hand, the change of depth from 0.5mm to 3mm less significant effect on the O_2 level.

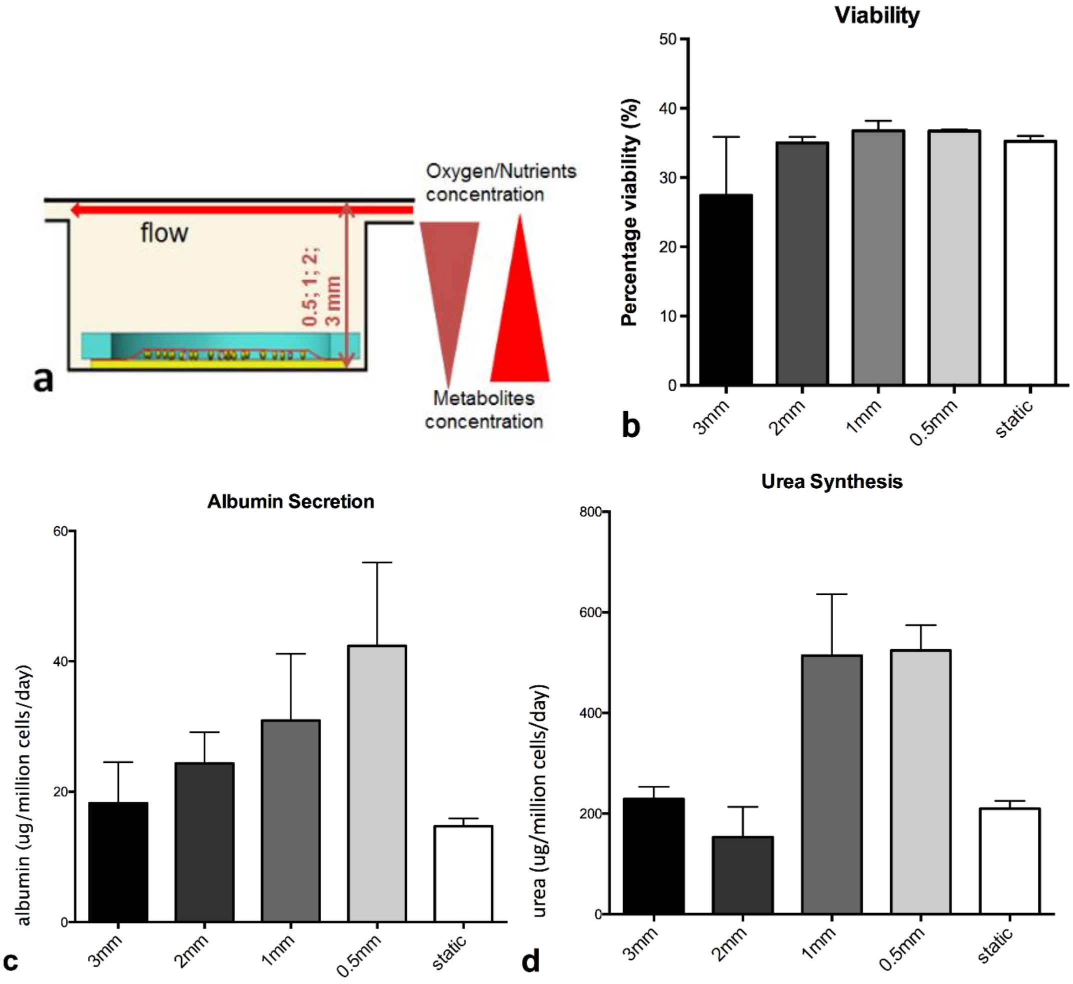

Figure S3: a) Schematic design with the positioning of the CS model in the microwell; the concentration of the O_2 and nutrients decrease with the depth of the chamber culture. S3b) Cell viability was not significantly different for different chamber depths, cell number was normalized to the initial cell number. S3c) Albumin production was highest at 0.5mm d) urea synthesis was highest at 1 mm and 0.5 mm. Results were obtained from triplicate measurements (n=3), *p≤0.05. Data from representative experiments are presented, whereas similar trends were seen in multiple trials.

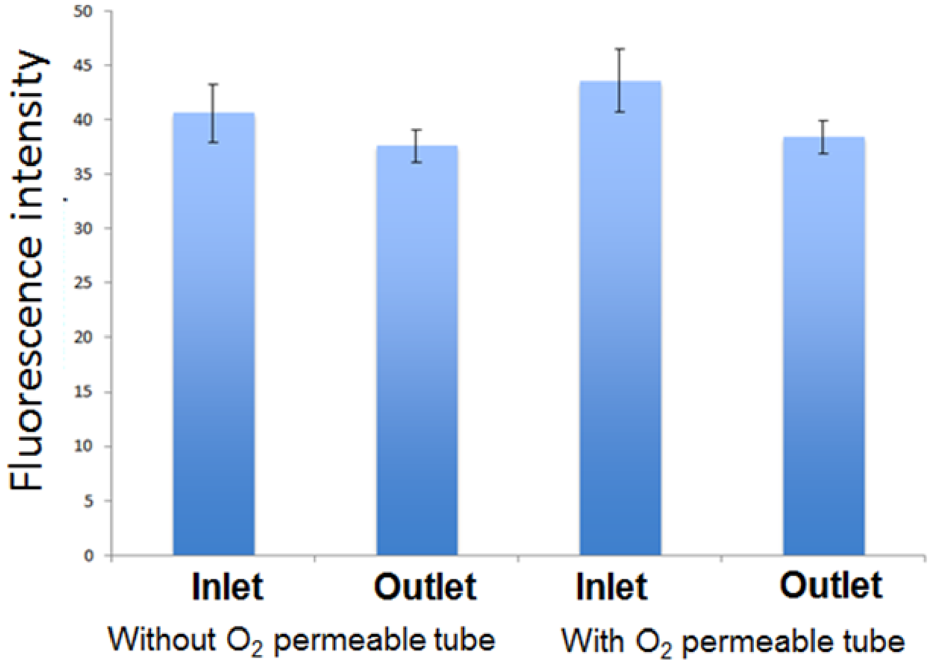

Figure S4: The drop in oxygen concentration in the media after flowing at 0.1mL/h through the PIC system loaded with rat hepatocytes spheroids (~2 x 10^4 cells) in the presence/absence of oxygen permeable tube

a

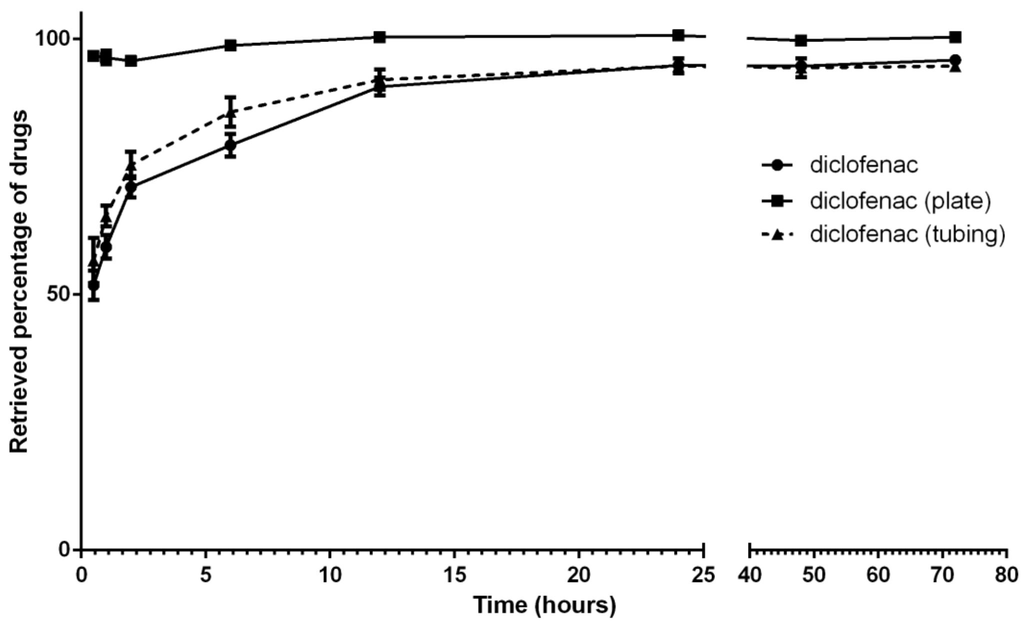

b

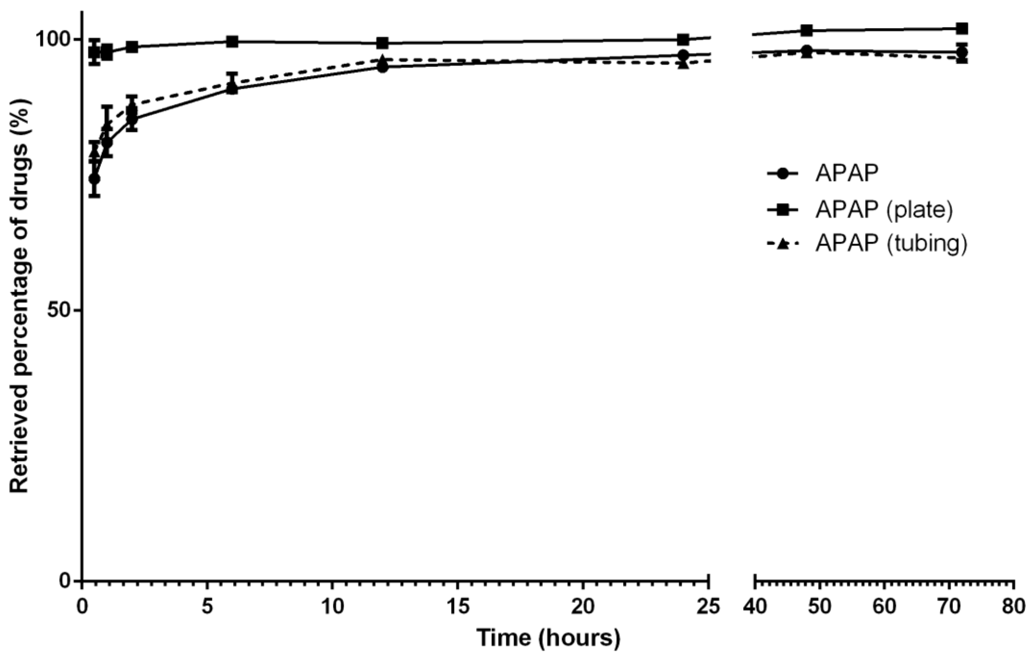

Figure S5: Amount of (a) diclofenac and (b) APAP retrieved after flowing through the PIC, tubing system (without PIC) and well-plate, normalized to the total amount of drug loaded into PIC, tubing and well-plate for 0.5 to 72 hours. Results are the average ± the standard error of the mean collected from 3 different experiments.
